# Seroprevalence of Hepatitis C in Ethiopia: First National Study Based on the 2016 Ethiopian Demographic and Health Survey

**DOI:** 10.1111/jvh.14037

**Published:** 2024-11-21

**Authors:** Getahun Molla Kassa, Atsbeha Gebreegziabxier Weldemariam, Saro Abdella Abrahim, Clare E. French, Dawit Wolday, Emebet Dagne, Andargachew Mulu, Aynishet Adane, Sarah K. Inglis, Andrew Radley, Geremew Tasew, Peter Vickerman, Elias Ali Yesuf, Ora Paltiel, Mesay Hailu, Wondwossen Amogne, John F. Dillon, Matthew Hickman, Aaron G. Lim, Josephine G. Walker, the DESTINE NIHR Global Health Research Group, John F. Dillon, Wondwossen Amogne, Peter Vickerman, Matthew Hickman, Ora Paltiel, Dawit Wolday, Aynishet Adane, Saro Abdella, Zenahbezu Abay, Workagegnehu Hailu, Tadesse Awoke, Emebet Dagne, Elias Ali Yesuf, Josephine G. Walker, Aaron G. Lim, Clare E. French, Andargachew Mulu, Melaku Tileku Tamiru, Atsbeha Gebreegziabxier Weldemariam, Christie Cabral, Obsie Baissa, Elizabeth Speakman, Andrew Radley, Amy Malaguti, Sarah K. Inglis, Meseret Yohannes, Bruktait Taddele, Hagos Abraha, Mengistu Erkie, Tesfa Sewunet Alamneh, Getahun Molla Kassa

**Affiliations:** ^1^ Population Health Sciences, Bristol Medical School University of Bristol Bristol UK; ^2^ Department of Epidemiology and Biostatistics, Institute of Public Health, College of Medicine and Health Sciences University of Gondar Gondar Ethiopia; ^3^ Ethiopian Public Health Institute Addis Ababa Ethiopia; ^4^ Division of Molecular and Clinical Medicine, School of Medicine University of Dundee Dundee UK; ^5^ Health Laboratory Services Ethiopian Public Health Institute Addis Ababa Ethiopia; ^6^ NIHR Health Protection Research Unit in Behavioural Science and Evaluation at University of Bristol Bristol UK; ^7^ Department of Biochemistry & Biomedical Sciences McMaster University Hamilton Ontario Canada; ^8^ Department of Internal Medicine, Institute of Health Jimma University Jimma Ethiopia; ^9^ Armauer Hansen Research Institute Addis Ababa Ethiopia; ^10^ Department of Internal Medicine, School of Medicine, College of Medicine and Health Sciences University of Gondar Gondar Ethiopia; ^11^ Tayside Clinical Trials Unit University of Dundee Dundee UK; ^12^ Division of Public Health and Genomics, School of Medicine University of Dundee Dundee UK; ^13^ Department of Health Policy and Management, Institute of Health Jimma University Jimma Ethiopia; ^14^ Department of Hematology and Braun School of Public Health Hadassah‐Hebrew University Faculty of Medicine Jerusalem Israel; ^15^ Department of Internal Medicine, School of Medicine, College of Health Sciences Addis Ababa University Addis Ababa Ethiopia

**Keywords:** EDHS, Ethiopia, HCV, seroprevalence, survey

## Abstract

Hepatitis C virus (HCV) is hypothesised to be a public health problem in Ethiopia, and systematic review evidence suggested 1%–3% seroprevalence. We aimed to estimate the seroprevalence of HCV overall and across regions of Ethiopia. We estimated HCV seroprevalence using the 2016 Ethiopian Demographic and Health Survey (EDHS‐2016). EDHS‐2016 is a nationwide household survey conducted using two‐stage cluster sampling methods. We tested all 26,753 samples from participating adult women (15–49 years) and men (15–59 years) using HCV Enzyme Immunoassay. Descriptive analyses were performed based on the Guide to Demographic Health Survey statistics. We applied sample weighting to derive representative estimates. Of the total tested, more than half (54.40%) were aged 15–29 years and 51.59% were women. Overall HCV seroprevalence was 0.18% (95% Confidence Interval: 0.10–0.32). Higher seroprevalences were found in Afar (0.92%) and South Nations Nationality Peoples Region (0.43%); people living with HIV (PLWH) (0.62%); the poorest wealth index (0.35%); people having multiple lifetime sexual partners (0.31%); and widowed/divorced individuals (0.30%). In stratified analyses by sex and residency, we found higher seroprevalences in non‐Christian and non‐Muslim males (1.98%) and rural population (1.00%), male PLWH (1.67%), rural PLWH (1.45%), widowed/divorced males (0.97%), and in all groups from the Afar region: males (1.30%), females (0.61%), urban (1.07%), and rural (0.86%). HCV seroprevalence among the general population in Ethiopia is much lower than from previous estimates. General population screening is unlikely to be cost‐effective, and so screening programs targeted to people at greater risk of HCV will be required.

**Abbreviations**
Ab: antibody
CI: confidence interval
CIA: chemiluminescence assay
DBS: dried blood spot
DHS: demographic health survey
EAs: enumeration areas
EDHS: Ethiopian demographic and health survey
EIA: enzyme immunoassay
EPHI: Ethiopian public health institute
FGM: female genital mutilation/cutting
HCV: hepatitis C virus
HIV: human immunodeficiency virus
MoH: Ministry of Health‐Ethiopia
PLWH: people living with HIV
SNNPR: south nations nationality peoples region
STIs: sexual transmitted infections
WHO: World Health Organisations

## 1 Introduction

Hepatitis C virus (HCV) causes substantial morbidity and mortality and presents a global public health problem. Globally, 58 million people were estimated to be chronically infected with HCV, and 0.3 million died due to HCV‐related disease in 2019 [1]. The population affected by HCV varies across regions. However, sub‐populations such as people who inject drugs or sub‐groups with a history of unsafe medical procedures or unscreened blood transfusions have a particular high risk of infection [1].

The Ethiopian government recognised HCV as a public health problem in 2016 [2]. However, available studies from Ethiopia have produced inconsistent seroprevalence estimates. A recent systematic review by our group in 2023 estimated a pooled seroprevalence of 1.6% among the general population [3]. Similarly, a systematic review from 2022 using published data from 2010 to 2020 reported a pooled seroprevalence of 2%, with lower estimates in blood donors (1%) and higher estimates in people living with HIV (PLWH) (4%) and in mixed groups (5%) [4]. Conversely, an older systematic review from 2016 estimated a higher pooled seroprevalence of 3.1%, with a higher pooled seroprevalence among PLWH (5.5%) but slightly lower (2.4%) for the general population [5]. Both these systematic reviews pooled estimates from different risk populations, including liver disease patients. On the other hand, small geographically scattered community‐based studies estimated HCV seroprevalence ranging from 0.6% to 1.9% [6, 7, 8, 9, 10, 11, 12]. A nationwide biobehavioural survey in 2020 among female sex workers estimated a seroprevalence of 0.5% [13], while a similar survey among people who inject drugs in Addis Abba estimated a 2.9% HCV seroprevalence in 2015 [14].

The mode of HCV transmission in Ethiopia is not well known. Some of the reported risk factors in the literature are age at first sex ≤ 15, older age, risky sexual behaviour (practising sex work or buying sex), being HIV positive, history of blood transfusion or unsafe medical procedures, and traditional practices [8, 10, 13, 15, 16]. However, Ethiopia is a diverse nation with multiple practices that may lead to HCV transmission that are not well explored, such as unsafe health care practices, over‐the‐counter medical injections, tribal markings, female genital mutilations, and other traditional practices such as male circumcision, tooth extraction, tattooing, and barbering.

In 2016, the Ministry of Health‐Ethiopia (MoH) developed its first national viral hepatitis prevention and control strategic plan 2016–2020, aligned with the WHO Global Health Sector Strategy targets set by WHO for HCV, which include an 80% reduction in incidence and a 65% reduction in mortality by 2030 compared to 2015 [17, 18]. A midterm review of this strategic plan in 2018 indicated that the strategies had not entirely been implemented and recommended revising it by looking at available data. Based on this recommendation, the MoH developed a revised second strategic plan 2021–2025. Both strategic plans for highlighted the absence of nationally representative HCV prevalence data as the main barrier to reasonable resource allocations and planning and advocated for a national HCV surveillance program as soon as possible [2, 18]. Though the government recognised that viral hepatitis is a public health problem, the national viral hepatitis program remains weak, with limited allocated budget, limited services centralised in specialised hospitals only, and much less attention being given to hepatitis compared to HIV, tuberculosis, and malaria [2, 19].

There is no consensus on HCV seroprevalence estimates in Ethiopia. To strengthen the existing HCV epidemiological evidence, we used the 2016 Ethiopian Demographic and Health Survey (EDHS‐2016) stored dried blood spots (DBS) and survey dataset to determine the national HCV seroprevalence in Ethiopia and identify population sub‐groups at increased risk of exposure.

## 2 Methods and Materials

### 2.1 Survey Design

**FIGURE 1:**
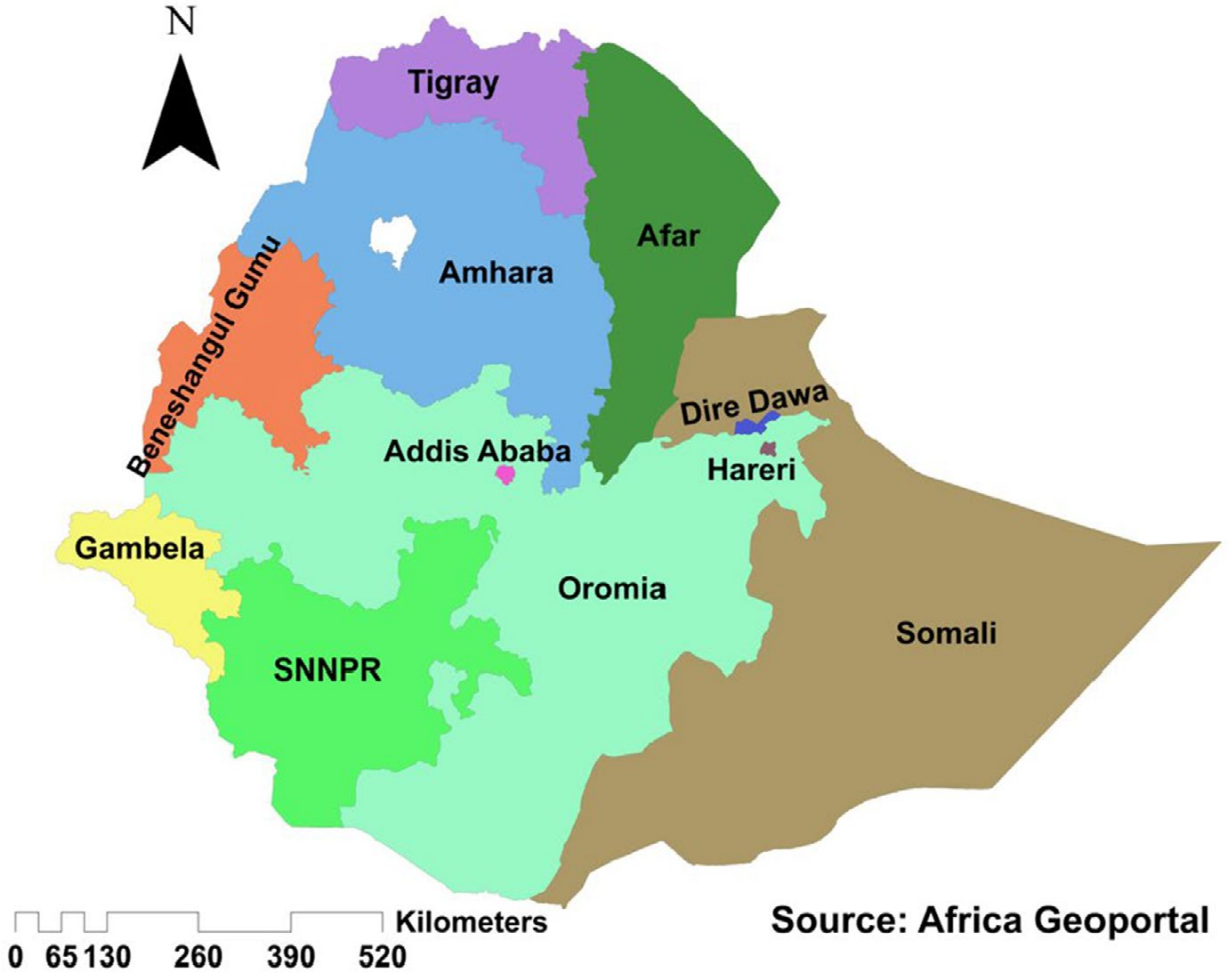
Map of Ethiopia with its regional administrative classification, 2016.

This analysis used the EDHS‐2016 stored DBS and survey dataset, which is a nationwide representative household survey conducted from January 18 to June 27, 2016. Demographic Health Surveys (DHS) are designed to provide a wide range of representative data in health and health‐related indicators, including disease prevalence at national, regional, and urban versus rural levels using population sociodemographic and economic characteristics [20]. At the time of the EDHS‐2016, the country had nine regional administrations (Tigray, Afar, Amhara, Oromia, Somali, Gambela, Benshangul‐Gumuz, Harari, and South Nations Nationality Peoples Region (SNNPR)) and two federal charter cities (Addis Ababa (capital city) and Dire Dawa) (see Figure 1). EDHS‐2016 used a two‐stage stratified cluster sampling method. The sampling frame was based on the 2007 Ethiopian population and housing census, which had a complete list of 84,915 enumeration areas (EAs) with location data and urban/rural residency. These EAs were stratified into urban/rural, and then, with a probability proportional selection method, 202 urban and 443 rural EAs were selected independently. In each selected cluster (EAs or segment of the EAs), 28 households were randomly selected, and all women aged 15–49 and men aged 15–59 who were permanent residents or visitors who stayed a night before the interview in the selected households were eligible to be interviewed. We used data from all interviewed women aged 15–49 and men aged 15–59 who gave blood for HIV testing. More details of the survey design are available within the EDHS‐2016 report [21] and a summary in Data S1.

### 2.2 Blood Sample Collection and HCV Antibody Testing

Blood from finger pricks was collected and smeared on five blood spots on a Whatman 903 filter paper card. For each participant, a unique barcode was applied on the sample card, the biomarker questionnaire, and the DBS transmission sheet used to track the blood sample from the field to the laboratory. During the survey, samples were collected, pasted on filter paper cards, dried overnight, and packed the next morning for transportation to the Ethiopian Public Health Institute (EPHI), Addis Ababa, where they were given a laboratory number (laboratory code), frozen stored at under −20°C, and used for testing. The quality of DBS samples stored for 7 years involves assessing their stability and integrity over long‐term storage based on the EPHI quality manual policy. The long‐term stability of HCV antibodies under these storage conditions has been validated in other studies [22, 23, 24].

We tested the DBS samples for HCV antibody in 2023 using the WHO pre‐qualified INNOTEST HCV Ab IV qualitative enzyme immunoassays (EIA) (Fujirebio Europe N.V. Ghent, Belgium), which has 100% sensitivity and specificity [25]; and a company claim of 100% sensitivity and 99.80% specificity [26]. To make testing more cost‐effective, a pooling technique was developed and validated for HIV‐negative samples; details are included in Data S2. Pooling experiments suggested four samples per pool were optimum for efficiency. The pooling of DBS samples was done for HIV‐negative individuals because it was assumed that the HCV prevalence would be lower in this sub‐population, and so firstly, pooling samples would reduce the number of tests that are used, with subsequent testing of all individuals in a pooled sample that tested positive. HIV‐positive samples were tested on individuals because we thought that the HCV prevalence might be higher and more of the blood spots had already been used for other HIV‐related tests. The samples were tested at EPHI by highly trained laboratory professionals supervised by a senior expert. We used comprehensive quality control and assurance measures such as detailed protocols, trained laboratory personnel, and maintained comprehensive records of all samples and regular positive and negative control checks. Detailed testing procedures are in Data S2.

### 2.3 Data Entry

We entered the HCV test result into the EDHS‐2016 HIV dataset using the sample barcode and laboratory code. And then linked to recoded household, female, and male datasets using a unique identifier (cluster number, household number, and respondent's line number).

### 2.4 Variables of the Interest

The outcome variable of interest was HCV seroprevalence, classified as positive or negative. We explored HCV seroprevalence variations for pre‐specified variables such as participants' age, sex, marital status, educational status, occupation, wealth index, religion, urban/rural residency, regional states, and available behavioural, epidemiological, and clinical characteristics such as age at first sex, sexual history, ever consumed alcohol, ever chewed khat, HIV status, self‐reported sexual transmitted infections (STIs), male circumcision or female genital mutilation (FGM) status, and whether the participant received medical injection in the last 12 months. The wealth index score was derived using principal component analysis based on the number and kind of goods they possess and their housing characteristics, after which the score was divided into quintiles (poorest, poorer, middle, richer, and richest) (see EDHS‐2016 report for details [21]).

### 2.5 Data Analyses

All data analyses were done using STATA/MP 17 software [27] based on the Guide to DHS statistics [20]. To ensure a representative result, we applied survey data weighting techniques in all our analyses. In the EDHS‐2016, there was non‐proportional allocation of samples to each regional state and EAs. There was also a difference in response rate at the household and individual level. The weighting factor was calculated by considering all sampling parameters, selection probabilities, and adjusted for nonresponse rate at the household and individual level as provided within the EDHS‐2016 HIV dataset. Details on the calculation of the weighting factor are available in the EDHS‐2016 report, annex A.4 [21]. We performed descriptive statistics for each population characteristic and estimated HCV seroprevalence overall and by population characteristics. We estimated HCV seroprevalence separately across the population characteristics for males, females, urban, and rural by anticipating that risk factors might be different across sex and urban/rural residency. We reported point seroprevalence with a 95% Confidence Interval (CI).

We planned to run a multilevel bivariable and multivariable binary logistic regression to assess risk factors and control the confounding effects of explanatory variables. However, due to the low number of HCV antibody‐positive samples (62/26,753), the chi‐square assumptions failed for most of the explanatory variables to run a regression analysis. Similarly, we also planned to run a spatial analysis to see the geographical clustering of HCV seroprevalence; again, because of this low number of HCV antibody‐positive samples, it was difficult to assess spatial clustering; however, we did assess the seroprevalence in different regions.

### 2.6 Ethics

The study subjects who provided blood for HIV tests consented to store the samples in the laboratory for future HCV, hepatitis B virus, rubella, and measles testing during the survey. The interviewer informed the participants that test results would not be provided back. If a study participant refused use of their stored sample for future testing, their refusal was documented on the biomarker questionnaire, and the word ‘no additional testing’ was printed on the filter paper card [21]. No DBS samples were tagged with ‘no additional testing’. Furthermore, we obtained a waiver of informed consent from the EPHI IRB to use the data and stored DBS samples for HCV seroprevalence (details are in Data S3).

## 3 Results

### 3.1 Sociodemographic Characteristics

All samples (26,753) tested for HIV were successfully tested for HCV antibodies. The median age of the study population tested for HCV was 28 years (inter quartile range: 15–36). In the weighted analysis, more than half (54.40%) were in the age group 15–29 years, and 51.59% were female. Over a third (37.51%) were below primary educational status. Nearly 80% lived in rural areas, and 16.37% were in the poorest wealth index. More than a quarter (28.88%) had a history of multiple sexual partners, and one‐fifth (19.16%) first had sex at or before age 15 years. Close to 1% (0.93%) were HIV infected, and 0.90% had self‐reported STIs in the past year (see Table 1).

**TABLE 1:** Weighted and unweighted study population sociodemographic characteristics and hepatitis C virus seroprevalence (%) in Ethiopia, 2016.

Variable	Categories	Unweighted *N* (%)	Weighted		
			%	*n*/*N*	HCV seroprevalence (%) (95% CI)
Overall		26,753 (100)		46/25,776	0.18 (0.10–0.32)
Sex	Male	11,968 (44.74)	48.41	28/12,479	0.23 (0.10–0.50)
	Female	14,785 (55.26)	51.59	18/13,297	0.13 (0.07–0.25)
Current age in years	15–29	14,834 (55.45)	54.40	20/14,023	0.14 (0.07–0.30)
	30–39	6738 (25.19)	25.81	13/6654	0.20 (0.09–0.41)
	40–49	4117 (15.39)	15.54	10/ 4005	0.24 (0.10–0.60)
	50–59 (males)	1064 (3.98)	4.25	3/1094	0.27 (0.05–1.55)
Highest education level attained	No/preschool/don't know	9828 (36.73)	37.64	26/9702	0.27 (0.13–0.58)
	Primary	10,356 (38.71)	42.58	16/10,974	0.15 (0.08–0.28)
	Secondary	4141 (15.48)	13.11	2.06/3380	0.06 (0.02–0.17)
	Higher	2430 (9.08)	16.67	1.31/1720	0.08 (0.01–0.55)
Current marital status	Never married	8522 (31.85)	31.18	7/8036	0.09 (0.04–0.20)
	Married	16,172 (60.45)	62.15	33/16,019	0.21 (0.11–0.38)
	Widowed/Divorced	2059 (7.70)	6.67	5/1720	0.30 (0.11–0.81)
Residency	Urban	8174 (30.55)	20.98	5/5407	0.10 (0.04–0.21)
	Rural	18,579 (69.45)	79.02	41/20369	0.20 (0.10–0.38)
Region	Tigray	2974 (11.12)	6.75	3/1739	0.19 (0.06–0.56)
	Afar	1789 (6.69)	0.76	2/197	0.92 (0.47–1.78)
	Amhara	3522 (13.16)	24.54	4/6326	0.06 (0.02–0.24)
	Oromia	3460 (12.93)	36.91	9/9513	0.10 (0.03–0.30)
	Somalia	2174 (8.13)	2.77	0.12/713	0.02 (0.00–0.13)
	Benishangul‐Gumuz	1975 (7.38)	1.01	1/261	0.30 (0.10–0.91)
	SNNPR	3345 (12.50)	20.72	24/5341	0.44 (0.16–1.21)
	Gambela	1860 (6.95)	0.28	0.18/73	0.24 (0.08–0.75)
	Harari	1270 (4.75)	0.25	0/64	0
	Addis Ababa	2699 (10.09)	5.43	3/1400	0.20 (0.07–0.57)
	Dire Dawa	1685 (6.30)	0.57	0.13/148	0.09 (0.01–0.59)
Religion	Christian	15,730 (61.03)	67.37	28/17,365	0.16 (0.07–0.38)
	Muslim	9783 (37.95)	31.30	15/8068	0.19 (0.09–0.39)
	Non‐Christian and non‐Muslim	263 (1.02)	1.33	3/343	0.94 (0.13–0.62)
Occupation	Not working	8818 (34.21)	29.43	9/7585	0.11 (0.04–0.31)
	Health Professional	176 (0.68)	0.41	1/107	1.23 (0.17–8.51)
	Other Professional	16,782 (65.11)	70.16	36/18,084	0.20 (0.10–0.38)
Age at first sex	Not had sex	6487 (25.17)	26.29	8/6777	0.12 (0.05–0.26)
	≤ 15	5203 (20.19)	19.16	10/4938	0.20 (0.08–0.49)
	> 15	14,086 (54.65)	54.55	28/14,061	0.20 (0.09–0.44)
If had sex, total lifetime number of sexual partner	One partner	11,894 (61.66)	60.82	15/11,555	0.13 (0.04–0.40)
	More than one partner	7395 (38.34)	39.18	23/7444	0.31 (0.18–0.54)
If had sex, sexually active in last 4 week	Yes	12,618 (65.42)	71.86	30/13,653	0.22 (0.11–0.42)
	No	6671 (34.58)	28.14	8/5346	0.14 (0.07–0.31)
Number of medical injections in the last 12 months	None	17,245 (66.90)	68.51	35/17,659	0.20 (0.11–0.35)
	One	2763 (10.72)	9.40	3/2424	0.11 (0.03–0.41)
	More than one	5768 (22.38)	22.09	8/5694	0.15 (0.05–0.47)
Ever alcohol drink	Yes	9963 (38.88)	40.71	13/10,453	0.13 (0.04–0.24)
	No	15,664 (61.12)	59.29	31/15,227	0.20 (0.09–0.46)
Ever khat chewed	Yes	4740 (18.50)	19.40	12/4981	0.24 (0.10–0.57)
	No	20,887 (81.50)	80.60	32/20,699	0.16 (0.07–0.33)
Male circumcision or FGM	Yes	15,022 (83.80)	84.61	32/15,760	0.21 (0.11–0.40)
	No	2866 (15.99)	15.23	5/2836	0.17 (0.06–0.50)
	Don't know	37 (0.21)	0.17	0/31	0
If yes, who performed circumcision/FGM	Health Professional	8284 (55.15)	57.74	15/9100	0.16 (0.08–0.32)
	Traditional/Don't know	6738 (44.85)	42.26	18/6660	0.27 (0.11–0.66)
Wealth Index	Poorest	6513 (24.34)	16.37	15/4219	0.35 (0.16–0.79)
	Poorer	3772 (14.10)	18.20	7/4690	0.16 (0.05–0.48)
	Middle	3646 (13.63)	19.19	10/4946	0.20 (0.08–0.55)
	Richer	3879 (14.50)	20.53	8/5292	0.16 (0.07–0.37)
	Richest	8943 (33.43)	25.72	5/6630	0.08 (0.04–0.17)
HIV status	Positive	408 (1.53)	0.93	2/239	0.62 (0.12–3.12)
	Negative	26,339 (98.45)	99.06	44/25,534	0.17 (0.09–0.32)
	Unknown	6 (0.02)	0.01	0/3	0
Self‐reported STIs in the last 12 months	Yes	200 (0.78)	0.90	0/232	0
	No	25,564 (99.18)	99.06	46/25,533	0.18 (0.10–0.32)
	Don't know	12 (0.05)	0.04	0/11	0

Abbreviations: CI: confidence interval; FGM: female genital mutilation; HIV: human immunodeficiency virus; *n*: number of HCV antibody positives; *N*: number of HCV antibody tested; SNNPR: South Nations Nationality Peoples Region; STIs: sexually transmitted infections.

### 3.2 HCV Seroprevalence

**FIGURE 2:**
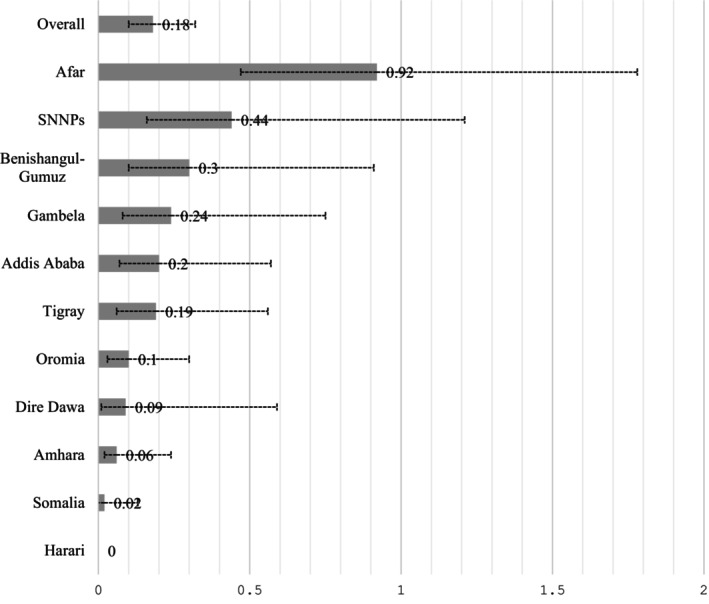
Weighted HCV seroprevalences (%) with 95% confidence intervals for each regional state and overall Ethiopia, 2016.

Of the 26,753 samples tested for HCV antibody, 62 were antibody‐positive (ever infected) (detailed, unweighted results are available in Data S4). When weighted, the overall sample size and the number of HCV antibody positives were adjusted to 25,776 and 46, respectively. This resulted in an overall weighted HCV seroprevalence of 0.18% (95% CI: 0.10–0.32). Regionally, the highest seroprevalence was documented in Afar (0.92%; 95% CI: 0.47–1.78) and SNNPR (0.44%; 95% CI: 0.16–1.21) (Figure 2). PLWH (0.62%; 95% CI: 0.12–3.12), individuals from the poorest wealth index (0.35%; 95% CI: 0.16–0.79), individuals with more than one lifetime sexual partner (0.31%; 95% CI: 0.18–0.54), and widowed/divorced persons (0.30%; 95% CI: 0.11–0.81) had higher seroprevalences than other population sub‐groups. There was weak evidence of higher seroprevalence in males (0.23%; 95% CI: 0.10–0.50) than females (0.13%; 95% CI: 0.07–0.25) and in rural (0.20%; 95% CI: 0.10–0.38) than urban populations (0.10%; 95% CI: 0.04–0.21) but with notable overlap in the 95% CI. Due to the low number of seropositive and small sample sizes for most explanatory variables, such as health professionals, there was considerable uncertainty in the seroprevalence estimates (Table 1).

### 3.3 HCV Seroprevalence Stratified by Respondent's Sex and Urban/Rural Residency

The difference in HCV seroprevalence between male vs. female and urban versus rural population was not statistically significant. However, the highest seroprevalences were found among males with any of the following characteristics: non‐Christian and non‐Muslim (1.98%), PLWH (1.67%), widowed/divorced marital status (0.97%), living in SNNPR regions (0.74%), or in the poorest wealth index (0.58%). Conversely, for females, with considerable uncertainty, health professionals had the highest seroprevalence (2.59%), but the sample size is very small (1/107). When looking across urban and rural populations, urban health professionals (1.69%) and rural individuals with non‐Christian and non‐Muslim (1.00%) or that are PLWH (1.45%) had elevated seroprevalence. The highest seroprevalences in all groups were for the Afar region compared with other regions: males (1.30%), females (0.61%), urban (1.07%), and rural (0.86%). There was considerable uncertainty and overlap across groups in the seroprevalence estimates due to the low number of seropositives (Table 2).

**TABLE 2:** Weighted HCV seroprevalence (%) stratified by respondent's sex and residency across population characteristics, 2016.

Variable	Categories	Weighted HCV seroprevalence (%) (95% CI)			
		Male	Female	Urban	Rural
Sex	Male	na	na	0.04 (0.01–0.15)	0.27 (0.12–0.61)
	Female	na	na	0.14 (0.06–0.36)	0.13 (0.06–0.28)
Current age in year	15–29	0.11 (0.03–0.37)	0.17 (0.08–0.36)	0.08 (0.03–0.27)	0.16 (0.07–0.37)
	30–39	0.34 (0.15–0.79)	0.08 (0.02–0.31)	0.04 (0.01–0.32)	0.23 (0.11–0.50)
	40–49	0.38 (0.13–1.12)	0.09 (0.03–0.24)	0.24 (0.09–0.65)	0.24 (0.08–0.72)
	50–59 (males)	0.27 (0.05–1.55)	na	0.15 (0.02–1.10)	0.30 (0.04–2.03)
Religion	Christian	0.20 (0.06–0.65)	0.12 (0.06–0.24)	0.09 (0.03–0.25)	0.18 (0.07–0.49)
	Muslim	0.21 (0.08–0.57)	0.17 (0.05–0.53)	0.12 (0.04–0.34)	0.20 (0.09–0.44)
	Non‐Christian and non‐Muslim	1.98 (0.30–12.09)	0	0	1.00 (0.14–6.73)
Occupation	Not working	0.05 (0.02–0.16)	0.12 (0.04–0.35)	0.05 (0.01–0.22)	0.13 (0.04–0.38)
	Health professional	0	2.59 (0.35–16.76)	1.69 (0.22–11.68)	0
	Other professional	0.24 (0.11–0.54)	0.12 (0.06–0.26)	0.08 (0.03–0.21)	0.23 (0.11–0.46)
Highest education level attained	No/preschool/don't know	0.49 (0.18–1.38)	0.14 (0.05–0.39)	0.06 (0.01–0.28)	0.29 (0.13–0.62)
	Primary	0.17 (0.08–0.35)	0.12 (0.05–0.29)	0.13 (0.05–0.31)	0.15 (0.08–0.31)
	Secondary	0.03 (0.01–0.21)	0.10 (0.03–0.34)	0.08 (0.02–0.28)	0.04 (0.01–0.28)
	Higher	0	0.18 (0.03–1.31)	0.10 (0.01–0.72)	0
Current marital status	Never married	0.11 (0.04–0.31)	0.06 (0.02–0.17)	0.08 (0.03–0.22)	0.09 (0.03–0.26)
	Married	0.26 (0.12–0.56)	0.17 (0.08–0.34)	0.07 (0.02–0.33)	0.23 (0.13–0.44)
	Widowed/Divorced	0.97 (0.27–3.42)	0.10 (0.03–0.31)	0.33 (0.12–0.91)	0.29 (0.07–1.11)
Regional states	Tigray	0.20 (0.03–1.34)	0.18 (0.05–0.56)	0.10 (0.01–0.78)	0.21 (0.06–0.72)
	Afar	1.30 (0.61–2.76	0.61 (0.19–1.90)	1.07 (0.38–3.00)	0.86 (0.36–2.00)
	Amhara	0	0.12 (0.03–0.48)	0	0.07 (0.02–0.29)
	Oromia	0.12 (0.03–0.48)	0.08 (0.01–0.53)	0	0.11 (0.04–0.35)
	Somalia	0	0.03 (< 0.01–0.23)	0.09 (0.01–0.67)	0
	Benishangul‐Gumuz	0.17 (0.02–1.15)	0.43 (0.16–1.15)	0	0.36 (0.12–1.08)
	SNNPR	0.74 (0.25–2.17)	0.17 (0.06–0.53)	0.21 (0.02–1.72)	0.47 (0.16–1.37)
	Gambela	0.23 (0.07–0.83)	0.25 (0.08–0.85)	0.10 (0.01–0.77)	0.37 (0.10–1.36)
	Harari	0	0	0	0
	Addis Ababa	0.09 (0.01–0.66)	0.28 (0.08–0.97)	0.20 (0.07–0.57)	—
	Dire Dawa	0.18 (0.03–1.21)	0	0	0.29 (0.04–1.87)
Number of medical injections in the last 12 months	No	0.21 (0.09–0.45)	0.19 (0.10–0.37)	0.14 (0.06–0.34)	0.21 (0.11–0.40)
	One	0.17 (0.03–0.90)	0.06 (0.01–0.36)	0	0.16 (0.05–0.58)
	More than one	0.33 (0.09–1.18)	0.02 (0.01–0.08)	0.03 (0.01–0.24)	0.18 (0.05–0.61)
Age at first sex in years	Not had sex	0.12 (0.04–0.38)	0.11 (0.04–0.30)	0.16 (0.05–0.51)	0.10 (0.04–0.29)
	≤ 15	0.04 (0.01–0.19)	0.22 (0.09–0.54)	0.14 (0.04–0.51)	0.20 (0.08–0.56)
	> 15	0.28 (0.13–0.63)	0.08 (0.03–0.25)	0.05 (0.02–0.16)	0.24 (0.11–0.55)
If had sex, total lifetime number of sexual partner	One partner	0.17 (0.03–1.02)	0.11 (0.04–0.30)	0.03 (0.01–0.14)	0.15 (0.05–0.48)
	More than one partner	0.34 (0.17–0.69)	0.23 (0.09–0.60)	0.11 (0.04–0.30)	0.37 (0.20–0.68)
If had sex, is sexually active in last 4 week	Yes	0.27 (0.12–0.63)	0.17 (0.08–0.39)	0.03 (0.01–0.16)	0.26 (0.13–0.50)
	No	0.26 (0.10–0.68)	0.06 (0.02–0.15)	0.13 (0.05–0.33)	0.15 (0.06–0.39)
Male circumcision or FGM	Yes	0.22 (0.10–0.45)	0.18 (0.08–0.41)	0.09 (0.04–0.20)	0.24 (0.11–0.49)
	No	0.36 (0.09–1.42)	0.07 (0.02–0.28)	0	0.23 (0.08–0.68)
If yes, who performed the circumcision/FGM	Health Professional	0.16 (0.08–0.32)	0	0.05 (0.01–0.21)	0.19 (0.09–0.39)
	Traditional/don't know	0.42 (0.12–1.40)	0.18 (0.08–0.41)	0.14 (0.05–0.37)	0.30 (0.11–0.82)
Ever alcohol drink	Yes	0.10 (0.04–0.27)	0.16 (0.07–0.36)	0.11 (0.04–0.30)	0.13 (0.06–0.28)
	No	0.31 (0.11–0.86)	0.12 (0.05–0.29)	0.08 (0.02–0.30)	0.23 (0.09–0.55)
Ever chewed khat	Yes	0.25 (0.10–0.66)	0.22 (0.03–0.16)	0.10 (0.02–0.42)	0.27 (0.11–0.69)
	No	0.20 (0.07–0.60)	0.12 (0.07–0.22)	0.10 (0.04–0.24)	0.17 (0.07–0.41)
Wealth Index	Poorest	0.58 (0.22–1.56)	0.15 (0.04–0.50)	0	0.37 (0.16–0.83)
	Poorer	0.21 (0.07–0.62)	0.11 (0.03–0.46)	0	0.16 (0.05–0.49)
	Middle	0.19 (0.05–0.77)	0.21 (0.05–0.88)	0	0.21 (0.08–0.55)
	Richer	0.23 (0.09–0.62)	0.08 (0.01–0.51)	0	0.16 (0.07–0.38)
	Richest	0.03 (0.01–0.12)	0.12 (0.05–0.30)	0.11 (0.05–0.23)	0
HIV status	Positive	1.67 (0.24–10.74)	0.15 (0.04–0.60)	0.16 (0.04–0.66)	1.45 (0.20–9.52)
	Negative	0.22 (0.10–0.49)	0.13 (0.07–0.25)	0.10 (0.04–0.22)	0.19 (0.10–0.38)
	Unknown	—	0	0	0
Self‐reported STIs in the last 12 months	Yes	0	0	0	0
	No	0.23 (0.10–0.50)	0.13 (0.07–0.25)	0.10 (0.04–0.22)	0.20 (0.10–0.39)
	Don't know	0	0	0	0

Abbreviations: FGM: female genital mutilation; HIV: human immunodeficiency virus; na: not applicable; SNNPR: South Nations Nationality Peoples Region; STIs: sexually transmitted infections.

## 4 Discussion

In this first nationwide HCV seroprevalence survey in Ethiopia, we found a low overall HCV seroprevalence (0.18%). We observed variations across regions and population characteristics. There were comparatively higher HCV seroprevalence rates in the Afar (0.92%) and SNNPR (0.44%) regions, among PLWH (0.62%), and the deprived communities, but little difference between males and females overall.

This overall seroprevalence is much lower than found in previous systematic reviews by Deress et al. 2022 (2%) and Belyhun et al. 2016 (3.1%) [4, 5]. It is also lower than general population seroprevalence estimates of 2.4% by Belyhun et al. in 2016 [5] and 1.6% from a recent systematic review [3]. More similar seroprevalence estimates were found in two analyses of nationwide blood donor data (0.5% and 0.4%) [28, 29], although the representativeness of these datasets is hard to assess since blood donation in Ethiopia is either voluntary or replacement for stored blood by family/friends of the recipient, and there are donor selection criteria such as age above 18 years, absence of transfusion transmissible diseases like HCV, and other clinical conditions [30]. Unexpectedly, our seroprevalence is comparable to the HCV seroprevalence found in a recent nationwide biobehavioural survey among female sex workers (0.5%) [13]. The high HCV seroprevalence estimates from the two published systematic reviews are expected since they included studies from high‐risk populations such as people with liver disease, those on haemolysis, health care workers, clinic/hospital outpatients and inpatients, medical waste handlers, prisoners, refugees, PLWH, and psychiatric patients [4, 5]. All the reviews included studies from many different sites in the country and studies that used highly sensitive but low‐specificity HCV tests. In the most recent published systematic review (from 2022) [4], nearly half of included studies (44.64%, 25/56 studies) were from the Amhara region, 16.07% (9/56) from the SNNPR region, and there were no included studies from the Afar and Benishangul‐Gumuz regions, which indicates this estimate is not representative of the country. In our recent review, most included studies (55%; 6/11) are from Addis Ababa [3]. On the other hand, the low HCV seroprevalence estimates (0.4%–0.5%) among blood donors and female sex workers suggest this low seroprevalence might be a true estimate [13, 28, 29].

In line with our findings, two systematic reviews reported the highest HCV seroprevalences among PLWH [4, 5]. Similarly, other small‐scale studies reported that people with risky sexual behaviours [10, 16] and widowed/divorced women have higher HCV seroprevalence [31, 32].

Despite the low seroprevalence (0.18%), given that the Ethiopian adult population in 2023 is estimated to be 73.4 million [33], this seroprevalence estimate still suggests that approximately 132,000 adult persons have been exposed to HCV infection, and so about 92,000 adults should be chronically infected with HCV in Ethiopia (assuming 70% of HCV infected persons develop chronic HCV infections [34]). The regional and demographic variations suggested that HCV risk factors differ across geographical locations and population characteristics.

### 4.1 Strengths and Limitations

This study is the first to produce a national HCV seroprevalence estimate for Ethiopia using a large representative national sample, with the study highlighting specific sub‐populations that may have higher HCV exposure.

However, as with all studies, there are several limitations. In this study, using DBS samples stored for 7 years was used as the substrate for testing. Though DBS offers a wide range of benefits, it does have limitations related to the small sample volume it provides, the hemocrit effect, and variability in the quality and quantity of blood absorbed on the DBS card, all of which limit the number of samples suitable for testing and the number of tests [35, 41]. However, in this study the number of unsuitable samples was minimal, and there was no obvious biasing of unsuitable samples. Although the storage of the DBS samples can impact the stability of some biomarkers over time, especially without proper drying or storage [35], once dried, the DBS samples are stable, and so the impact of this is unlikely to affect the results.

Though it is recommended to use plasma/serum samples for INNOTEST HCV Ab IV EIA, the use of DBS samples instead of plasma/serum samples is an acceptable alternative to plasma for HCV antibody tests using INNOTEST HCV Ab IV EIA [36]. Two systematic reviews also concluded the use of DBS for HCV antibody test has comparable results with serum/plasma samples [37, 38], but none of them included papers that use INNOTEST HCV Ab IV EIA.

We acknowledge potential limitations in storing frozen samples for an extended period (7 years), which might result in degradation of HCV biomarkers, but there is currently no evidence to support degradation. Though the variabilities between inter assays need further evaluation (INNOTEST HCV Ab IV EIA vs. the rest of the following), there is evidence that indicated the detection of HCV antibodies did not differ for long‐term stored DBS up to 5 years compared to a recently prepared DBS using the VITROS anti‐HCV IgG chemiluminescence assay (CIA) and HCV 3.0 EIA, both from Ortho Clinical Diagnostics, Rochester, New York [22]. Two studies also reported that HCV antibodies for DBS stored under −20°C up to 200 days are stable with a minimum optic density difference [23, 24], although these two studies use different assays: Ortho HCV Version 2.0 ELISA [23], ETI‐AB‐HCVK‐4, DiaSorin (Saluggia, Vercelli, Italy), and HCV Ab, Radim (Pomezia, Roma, Italy) [24].

Sample pooling EIAs is recommended for HCV screening, with evidence suggesting it has comparative performance (little sensitivity loss) to testing each sample individually but has a marked cost saving [39, 40]. In this study, we used four sample pooling EIAs among HIV‐negative samples and individual EIAs for HIV‐positive samples, and HCV seroprevalence was low for both.

The low seroprevalence makes subset analysis of the data less reliable. We lacked power to detect differences between groups due to the low number of HCV Ab‐positives as the 95% CI overlapped each other, and some 95% CI were wide. Therefore, validating our seroprevalence estimates with further studies directed to high‐risk groups such as the Afar region and PLWH or using representative samples of the country's population, including the older population, is warranted. Furthermore, we were not able to assess the effect of some important risk factors for HCV transmission, such as injection drug use, unsafe blood transfusions, unsafe medical producers, and scarification, because the EDHS‐2016 survey did not collect information on these important behaviours.

## 5 Conclusion

Our findings suggest that HCV seroprevalence in Ethiopia is lower than previously thought, but the current estimate is more reliable than previous ones, reflecting the superior methodology used in the EDHS. The caveat to this is that high‐risk populations might be under‐represented in this survey and so require further study. However, given the large population of Ethiopia, HCV remains a significant treatable threat to the health of the nation, but the low HCV seroprevalence predicts that efforts to meet the WHO HCV elimination goals (80% reduction in incidence by 2030) might be feasible if appropriate public health responses are formulated, such as targeted screening and treatment for high‐risk groups as identified in this study.

## Author Contributions

A.G.L., J.G.W., C.E.F., M.H., G.M.K., E.A.Y., E.D., S.A.A., A.G.W., W.A., A.A., A.M., P.V., O.P., and J.F.D. conceptualised the study; A.G.L., J.G.W., M.H., E.A.Y., P.V., W.A., and J.F.D. acquisition fundings; S.K.I. managed the project overall; S.A.A. managed the sample storage, designed and wrote the proposal for testing the EDHS‐2016 samples for viral hepatitis, and obtained IRB clearance; A.G.W. developed novel laboratory methods and protocols to undertake pooled sample testing, conducted testing of the stored samples, and prepared the raw data, with support from S.A.A.; J.F.D., D.W., and S.A.A. supervised the sample testing in the laboratory; G.M.K. developed the epidemiological analysis methodology supported with A.G.L., C.E.F., J.G.W., and M.H.; G.M.K. performed the data linkage, cleaning, coding, formal analysis, interpretation, and visualisation of the data with support from A.G.L., J.G.W., C.E.F., M.H., E.A.Y., and E.D.; G.M.K. wrote the original draft manuscript and A.G.W. wrote the sample testing methods and sample storage protocols; A.R, G.T, contributed to the interpretation of results, provided comments on the manuscript, and approved the final version, as stated under the umbrella of all authors in the author contributions section. All authors interpreted the results, commented, and edited the manuscript; all authors approved the final version of the manuscript.

## Conflicts of Interest

The authors declare no conflicts of interest.

## DESTINE NIHR Global Health Research Group

Professor John F. Dillon (University of Dundee), Professor Wondwossen Amogne (Addis Ababa University), Professor Peter Vickerman (University of Bristol), Professor Matthew Hickman (University of Bristol), Professor Ora Paltiel (Hadassah‐Hebrew University), Dr. Dawit Wolday (Ethiopian Public Health Institute/ McMaster University), Dr. Aynishet Adane (University of Gondar), Mrs. Saro Abdella (University of Dundee/Ethiopian Public Health Institute), Dr. Zenahbezu Abay (University of Gondar), Dr. Workagegnehu Hailu (University of Gondar), Professor Tadesse Awoke (University of Gondar), Dr. Emebet Dagne (Jimma University), Dr. Elias Ali Yesuf (Jimma University), Dr. Josephine G. Walker (University of Bristol), Dr. Aaron G. Lim (University of Bristol), Dr. Clare E. French (University of Bristol), Dr. Andargachew Mulu (Armauer Hansen Research Institute), Melaku Tileku Tamiru (University of Dundee/Addis Ababa University), Atsbeha Gebreegziabxier Weldemariam (University of Dundee/Ethiopian Public Health Institute), Dr. Christie Cabral (University of Bristol), Dr. Obsie Baissa (Hadassah‐Hebrew University), Ms. Elizabeth Speakman (University of Dundee), Dr. Andrew Radley (NHS Tayside), Dr. Amy Malaguti (NHS Tayside), Dr. Sarah K. Inglis (University of Dundee), Ms. Meseret Yohannes (Addis Ababa University), Ms. Bruktait Taddele (Addis Ababa University), Dr. Hagos Abraha (Mekelle University), Dr. Mengistu Erkie (Addis Ababa University), Tesfa Sewunet Alamneh (University of Bristol/University of Gondar), Getahun Molla Kassa (University of Bristol/University of Gondar).

## Supporting information


Data S1–S4.

## Data Availability

The data that support the findings of this study are available on request from the corresponding author. The data are not publicly available due to privacy or ethical restrictions.
