# Supplementary material for: Seroprevalence of Hepatitis C in Ethiopia: First National Study Based on the 2016 Ethiopian Demographic and Health Survey
Source: J Viral Hepat. 2024 Nov 21;32(4):e14037. doi: 10.1111/jvh.14037 (PMC11883455; doi:10.1111/jvh.14037)
Supplement: Supplementary file 1 — Data S1–S4. [file JVH-32-0-s001.docx]

**Magnitude of Hepatitis C Virus in Ethiopia: *First National Seroprevalence Resulting from the 2016 Ethiopian Demographic and Health Survey***

Getahun Molla Kassa^1,2*†^, Atsbeha Gebreegziabxier Weldemariam^3,4^*, Saro Abdella Abrahim^5^, Clare E French^1,6^, Dawit Wolday^3,7^, Emebet Dagne^8^, Andargachew Mulu^9^, Aynishet Adane^10^, Sarah K Inglis^11^, Andrew Radley^12^, Geremew Tasew^3^, Peter Vickerman^1^, Elias Ali Yesuf^13^, Ora Paltiel^14^, Mesay Hailu^3^, Wondwossen Amogne^15^, John F Dillon^4^, Matthew Hickman^1^, Aaron G Lim^1^^, Josephine G Walker^1^^, on behalf of the DESTINE NIHR Global Health Research Group

**Authors Affiliations:**

^1^Population Health Sciences, Bristol Medical School, University of Bristol, Bristol, UK.

^2^Department of Epidemiology and Biostatistics, Institute of Public Health, College of Medicine and Health Sciences, University of Gondar, Gondar, Ethiopia.

^3^Ethiopian Public Health Institute, Addis Ababa, Ethiopia.

^4^Division of Molecular and Clinical Medicine, School of Medicine, University of Dundee, Dundee, UK

^5^Health Laboratory Services, Ethiopian Public Health Institute, Ethiopia

^6^ NIHR Health Protection Research Unit in Behavioural Science and Evaluation at University of Bristol, Bristol, UK.

^7^Department of Biochemistry & Biomedical Sciences, McMaster University, Hamilton, ON L8S 4K1, Canada.

^8^Department of Internal Medicine, Institute of Health, Jimma University, Jimma, Ethiopia.

^9^Armauer Hansen Research Institute, Addis Ababa, Ethiopia.

^10^Department of Internal Medicine, School of Medicine, College of Medicine and Health Sciences, University of Gondar, Gondar, Ethiopia.

^11^Tayside Clinical Trials Unit, University of Dundee, Dundee, UK

^12^Division of Public Health and Genomics, School of Medicine, University of Dundee, Dundee, UK.

^13^Department of Health Policy and Management, Institute of Health, Jimma University, Jimma, Ethiopia.

^14^Department of Hematology and Braun School of Public Health, Hadassah-Hebrew University Faculty of Medicine, Jerusalem, Israel.

^15^Department of Internal Medicine, School of Medicine, College of Health Sciences, Addis Ababa University, Addis Ababa, Ethiopia.

*Joint first author

^Joint senior author

^†^Corresponding Author

Getahun Molla Kassa

**Email**: [getahun.kassa@bristol.ac.uk](mailto:geteahun.kassa@bristol.ac.uk)

Bristol Medical School (Population Health Sciences), Oakfield House, Oakfield Grove, BS8 2BN, Bristol, United Kingdom**.**

**Appendixes**

**Appendix 1:** Summary of survey design

EDHS-2016 is a two-stage stratified cluster survey. The sampling frame was based on the 2007 Ethiopian population and housing census. First, EAs were stratified into urban and rural in each administrative region of the country. Then, with a probability proportional selection method, 202 urban and 443 rural EAs were selected independently. The list of households in each selected EA served as a sampling frame in the second sampling stage. Some large EAs were segmented into small areas to minimise the task of household listing, and one randomly selected segment will represent that EA. In each selected cluster (EAs or segment of the EAs) 28 households were randomly selected (see **Supplementary Figure 1)**.

**Supplementary Figure 1:** Summary of survey design

**Appendix 2: Summary of HCV antibody testing report.**

**2.1. Methods for validation of HCV DBS pooling sample (unpublished data)**

The validation of use of dried blood spot (DBS) sample pooling using the INNOTEST® HCV Ab IV, involves a series of steps to ensure that the method provides accurate and reliable results. The procedure was as follows:

1. Fresh DBS samples were collected for both HCV negative and positive individuals.

2. Pooling size was determined starting from 1 up to 10 DBS samples from individuals.

3. Antibodies were eluted from individual and pooled samples

4. ELISA was performed using INNOTEST® HCV Ab IV

5. The Analysis was calculated for sensitivity and specificity for individual and pooled samples.

6. Depending on the validation process, the pooling size was determined and accordingly four pool sizes were used for the HCV DBS serosurvey.

#### Therefore, Pooling DBS samples serve as an appropriate tool for use in large-scale screening of HCV.

#### 2.2. ELISA Testing procedures using INNOTEST® HCV Ab IV assay

ELISA Testing was performed at HIV and other viral disease serology testing laboratory, HIV and TB Research Directorate, Ethiopian Public Health Institute, Addis Ababa, Ethiopia. The ELISA testing was conducted according to manufacturer’s instructions, including the use of provided control materials for each run. The exception from the manufacturer’s instructions was that for the DBS eluates, no sample diluent was pre-loaded onto the plate (as it served as the elution buffer). For the DBS eluates, 200μl of each sample was loaded onto the EIA plate (equivalent to approximately 38 μl whole blood), and for plasma, 200μl sample diluent was pre-loaded and 20 μl plasma sample was added. By covering the plates with an adhesive sealer, the plate was incubated for 60 ± 3 minutes at 37 ± 1°C followed by washing each well 6 times using automated washer Machine. 200 µL conjugate working solution was added to each well and incubated for 60 + 3 minutes at 37 ±1°C followed washing each well 6 times using automated washer Machine. 200 µL substrate solutions was added to each well and incubated for 30 ± 1 minute at room temperature in the dark followed by adding 50 µL stop solution (sulfuric acid). The absorbance of the solution was read within 15 minutes at 450 nm with a microplate reader. The signal to cut-off ratio for the INNOTEST® HCV Ab IV assay was calculated according to manufacturer’s instructions (average of two positive optical density control measurements using a microplate reader with a 450 nm filter [OD_450_], divided by a factor of 2.75). There were no indeterminate or invalid results.

Between each sample, DBS punchers were cleaned by punching dry filter paper once, then four times with a filter saturated in 70% ethanol and to reduce cross contamination. Elution was carried out at room temperature with one hour of shaking at 500 rounds per minute, followed by an overnight incubation with no shaking and an additional hour of shaking at 500 rounds per minute.

**Principle**: Dried blood spot or dried blood sampling (DBS) is an innovative sampling technique where small blood samples are blotted on an absorbent paper and allowed to dry for analysis.

**2.3. ELISA Procedure for INNOTEST® HCV Ab IV using DBS samples**

All test materials must be brought to room temperature (18 to 30°C) approximately 30 minutes.

1. Take the strip-holder with the required number of strips, ensuring that for one strip, one SAM

control well, one negative and one positive control should be included; for more strips, at least

one SAM control well, two negative and two positive controls should be included in each strip

holder. During the test run, strips stay in the strip-holder and can be marked on one edge.

2. Punch one spot of the DBS samples and eluates with 315 μl sample diluent provided in the

INNOTEST® HCV Ab IV assay kit.

3. Add 200 µl of DBS eluates or control to each appropriate test well, except to the SAM

control well. A color change from purple to dark blue indicates that the specimen or control has

been added to the microwell. The SAM color change can also be read photometrically at a

wavelength of 620 nm:

 Blank the reader on the SAM control well according to the instrument manufacturer&#39;s

instructions.

 Each control or specimen should exhibit a value of greater than or equal to 0.100.

Make sure specimens and controls are adequately mixed with the Sample Diluent by pipetting up and down 5 times or by using a plate shaker at 1000 rpm for 1 minute.

4. Cover the strips with an adhesive sealer. Incubate for 60 ± 3 minutes at 37 ± 1°C.

NOTE: Prepare Conjugate Working Solution during incubation, see Reagents.

5. Wash each well 6 times (see Directions for washing).

6. Add 200 µl prepared Conjugate Working Solution to each well including the SAM control

well. A photometric read at a wavelength of 450 nm to document conjugate addition can be

performed after addition of Conjugate Working solution to the microwell strips:

 Do NOT blank the reader on the SAM control well.

 Each control or specimen should exhibit a value of greater than or equal to 0.950.

7. Cover the strips with a new adhesive sealer. Incubate for 60 ± 3 minutes at 37 ± 1°C.

NOTE: Prepare Substrate Solution during incubation.

8. Wash each well 6 times (see Directions for washing).

9. Add 200 µl prepared Substrate Solution to each well.

10. Incubate for 30 ± 1 minutes at room temperature in the dark.

11. To stop the reaction, add 50 µl Stop Solution to each well in the same sequence and at the

same time intervals as the Substrate Solution. Tap the strip holder carefully to ensure thorough

mixing.

12. Read the absorbance of the solution in the wells within 15 minutes after step 11 at 450 nm

with a microplate reader. Do NOT blank the reader on the SAM control well.

**Validation**

Check the validity of individual negative and positive controls (absorbances at 450 nm).

 Each of the negative controls should be lower than 0.100.

 Each of the positive controls should be higher than 0.800.

 Calculate P eliminating controls under 0.800.

If more than half the numbers of controls have to be eliminated, the test run should be repeated

after careful investigation into the source of errors.

Test result Calculate the cut-off value as: (P/2.75).

A sample is NON-REACTIVE if S < (P/2.75).

A sample is REACTIVE if S > (P/2.75).

IMPORTANT REMARK: - It is advised not to make a correction for a blank. This is because

samples which are borderline positive before correction can become borderline negative

afterwards. In this case, all OD values are lowered with the OD-value of the blank; the value of the cut-off is only lowered with the blank value divided by 2.75. A sample reactive upon initial testing must be retested in duplicate before results interpretation. A repeatedly reactive sample must be confirmed with an additional confirmatory test.

**Appendix 3:** **Ethics**

The study subjects who gave blood for HIV tests consented to store the blood samples in the laboratory for future HCV, hepatitis B virus, rubella, and measles testing during the survey. The interviewer explained to the survey participants about the procedure and the confidentiality of the data and informed the test results would not be available back. There are no personal identifiers or linked information in all the EDHS datasets, and the DHS public use dataset doesn’t allow respondents to be identified by any means. Furthermore, we obtained an ethical clearance letter from the Ethiopian Public Health Institute (EPHI) institutional ethics review board with reference number **EPHI 6.13/883** to test the stored blood samples for HCV. And we obtained a permission letter from the DHS programs to use the recoded EDHS-2016 household, woman, man, biomarker, and geographical coordinate datasets for our analysis.

**Appendix 4:** **Unweighted HCV Seroprevalence**

From 26,753 total DBS sample tested for HCV, 62 were anti-body positive and this makes the overall unweighted HCV seroprevalence of 0.23% (95% CI: 0.18-0.30) (**Supplementary Table 1)**.

**Supplementary Table 1**: Unweighted study population characteristics and hepatitis C virus seroprevalence (%) in Ethiopia, 2016.

| **Variable** | **Categories** | **Unweighted** | |
| --- | --- | --- | --- |
|  |  | **n/N** | **HCV Seroprevalence (95% CI)** |
| Overall |  | 62/26,753 | **0.23 (0.18-0.30)** |
| Sex | Male | 33/11,968 | 0.28 (0.20-0.39) |
|  | Female | 29/14,785 | 0.20 (0.14-0.28) |
| Current age in year | 15-29 | 30/ 14,804 | 0.20 (0.14-0.29) |
|  | 30-39 | 14/ 6,724 | 0.21 (0.12-0.35) |
|  | 40-49 | 14/ 4,103 | 0.34 (0.20-0.37) |
|  | 50 to 59 (males only) | 4/1,060 | 0.38 (0.14-1.00) |
| Highest education level attained | No education/ preschool | 29/9,750 | 0.30 (0.21-0.43) |
|  | Primary | 25/10,331 | 0.24 (0.16-0.36) |
|  | Secondary | 7/4,134 | 0.17 (0.08-0.35) |
|  | Higher to secondary | 1/2,429 | 0.04 (0.01-0.29) |
|  | Don’t know | 0/47 | 0 |
| Current marital status | Never married | 14/8,522 | 0.16 (0.10-0.28) |
|  | Married | 39/16,172 | 0.24 (0.18-0.33) |
|  | Widowed/Divorced | 9/2,059 | 0.44 (0.23-0.84) |
| Occupation | Not working | 15/8818 | 0.17 (0.10-0.28) |
|  | Health professionals | 1/176 | 0.57 (0.08-3.92) |
|  | Other professionals | 42/16,782 | 0.25 (0.19-0.34) |
| Wealth Index | Poorest | 28/6,513 | 0.43 (0.30-0.62) |
|  | Poorer | 8/3,772 | 0.21 (0.11-0.42) |
|  | Middle | 5/3,646 | 0.14 (0.06-0.33) |
|  | Richer | 7/3,879 | 0.18 (0.09-0.38) |
|  | Richest | 14/8,943 | 0.16 (0.09-0.26) |
| Residency | Urban | 13/8,174 | 0.16 (0.09-0.27) |
|  | Rural | 49/18,579 | 0.26 (0.20-0.35) |
| Region | Tigray | 6/2,974 | 0.20 (0.09-0.45) |
|  | Afar | 15/1,789 | 0.84 (0.51-1.39) |
|  | Amhara | 3/3,522 | 0.09 (0.03-0.26) |
|  | Oromia | 3/3,460 | 0.09 (0.03-0.27) |
|  | Somalia | 2/2,174 | 0.09 (0.02-0.37) |
|  | Benishangul-Gumuz | 6/1,975 | 0.30 (0.14-0.67) |
|  | SNNPs | 14/3,345 | 0.42 (0.25-0.71) |
|  | Gambela | 7/1,860 | 0.38 (0.18-0.79) |
|  | Harari | 0/1,270 | 0 |
|  | Addis Ababa | 5/2,699 | 0.19 (0.08-0.44) |
|  | Dire Dawa | 1/1,685 | 0.06 (0.01-0.42) |
| HIV status | Positive | 4/408 | 0.98 (0.37-2.58) |
|  | Negative | 58/26,339 | 0.22 (0.17-0.28) |
|  | Unknown | 0/6 | 0 |
| **Age at first sex** | Not had sex | 11/6,487 | 0.17 (0.09-0.31) |
|  | <15 | 16/5,203 | 0.31 (0.19-0.50) |
|  | >15 | 31/14,086 | 0.22 (0.15-0.31) |
| **Total lifetime number of sexual partner** | One partner | 21/11,894 | 0.18 (0.12-0.27) |
|  | Above one partner/don’t know | 26/7,395 | 0.35 (0.24-0.52) |
| **Recent sexual activity** | Last four weeks | 30/12,618 | 0.24 (0.17-0.34) |
|  | Not in last four weeks | 17/6,671 | 0.25 (0.16-0.41) |
| **Self-reported STI in the last 12 months** | Yes | 0/200 | 0 |
|  | No | 58/25,564 | 0.23 (0.18-0.29) |
|  | Don’t know | 0/12 | 0 |
| **Number of medical injections in the last 12 months** | None | 45/17,245 | 0.26 (0.20-0.35) |
|  | One injection | 5/2,763 | 0.18 (0.07-0.43) |
|  | Above one injection | 8/5,768 | 0.14 (0.07-0.28) |
| **Ever alcohol drink** | Yes | 20/9,963 | 0.20 (0.13-0.31) |
|  | No | 37/15,664 | 0.24 (0.17-0.33) |
| **Ever chewed khat/chat** | Yes | 12/4,740 | 0.25 (0.14-0.45) |
|  | No | 45/20,887 | 0.22 (0.16-0.29) |
| **Male circumcision or Female genital mutilation (FGM)** | Yes | 38/15,022 | 0.25 (0.18-0.35) |
|  | No | 7/2,866 | 0.24 (0.12-0.51) |
|  | Don’t know | 0/37 | 0 |
| **If yes, who performed circumcision/FGM** | Health Professionals | 17/8,284 | 0.21 (0.13-0.33) |
|  | Traditional/Don’t know | 21/6,738 | 0.31 (0.20-0.48) |

n: number of HCV seropositive; N: number of tested; CI: confidence interval; SNNPs: South Nations Nationality Peoples; HIV: human immunodeficiency virus.
